# Supplementary material for: New insights into US flood vulnerability revealed from flood insurance big data
Source: Nat Commun. 2020 Mar 19;11:1444. doi: 10.1038/s41467-020-15264-2 (PMC7081335; doi:10.1038/s41467-020-15264-2)
Supplement: Supplementary file 1 — Supplementary Information [file 41467_2020_15264_MOESM1_ESM.pdf]

New insights into US flood vulnerability revealed from flood insurance big data

SUPPLEMENTARY INFORMATION

O. E. J. Wing,[1,2] N. Pinter,[3,4] P. D. Bates,[1,2] C. Kousky[5]

[1]*School of Geographical Sciences, University of Bristol, Bristol, UK;* [2]*Fathom, Bristol, UK;* [3]*Department for Earth and Planetary Sciences, University of California, Davis, CA, US;* [4]*Center for Watershed Sciences, University of California, Davis, CA, US;* [5]*Wharton Risk Center, University of Pennsylvania, Philadelphia, PA, US*

SUPPLEMENTARY FIGURES

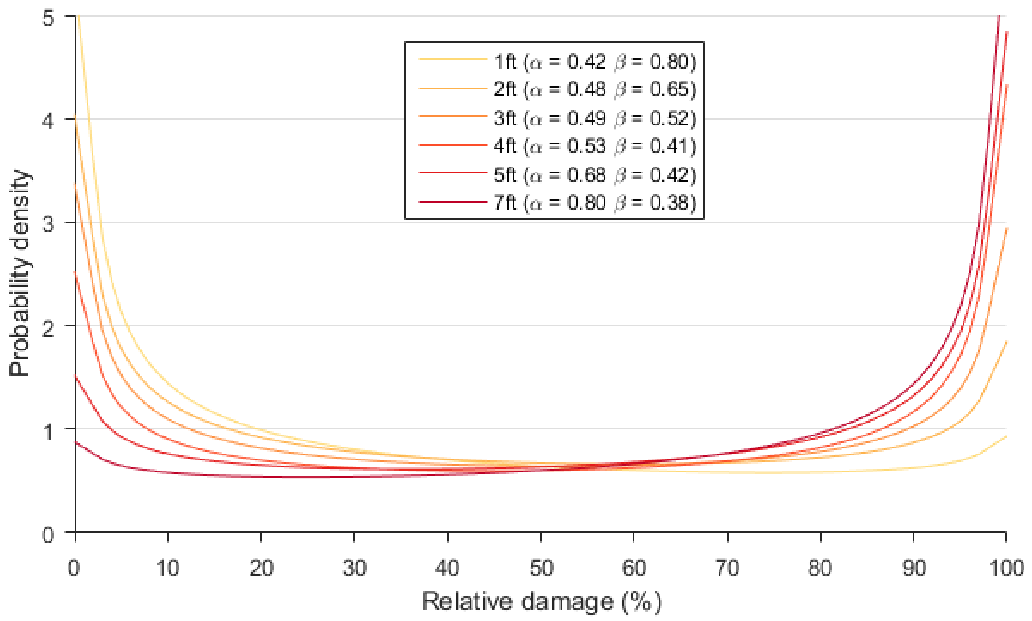

Supplementary Figure 1. Beta distributions of relative damage at reliably measured depth increments. Shape parameters α and β are indicated in the legend.

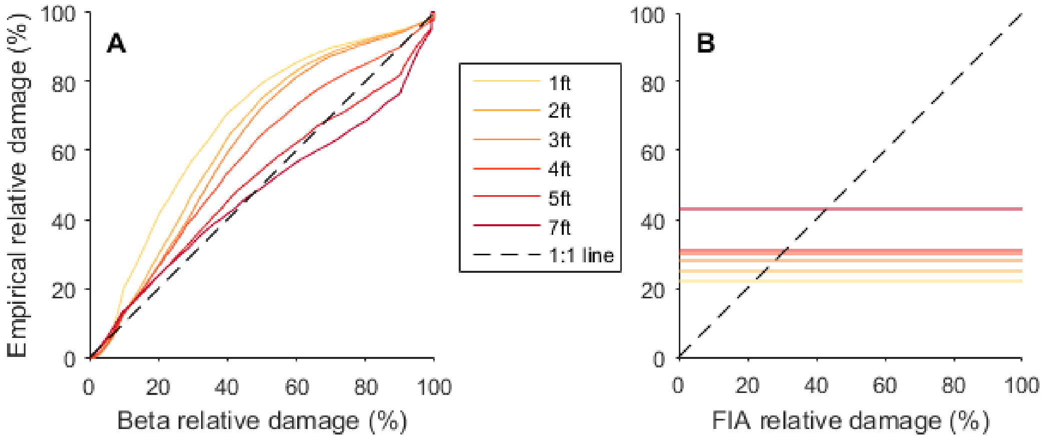

Supplementary Figure 2. Ranked quantiles of relative damage from the population of NFIP claims (*y* axis) vs. the corresponding quantile of relative damage from (**A**) samples from the

beta distributions (Supplementary Figure 1) and (**B**) the Federal Insurance Agency (FIA) depth–damage curve (*x* axis). Identical sample distributions would conform to the 1:1 line: R^2 values are shown in Supplementary Table 1.

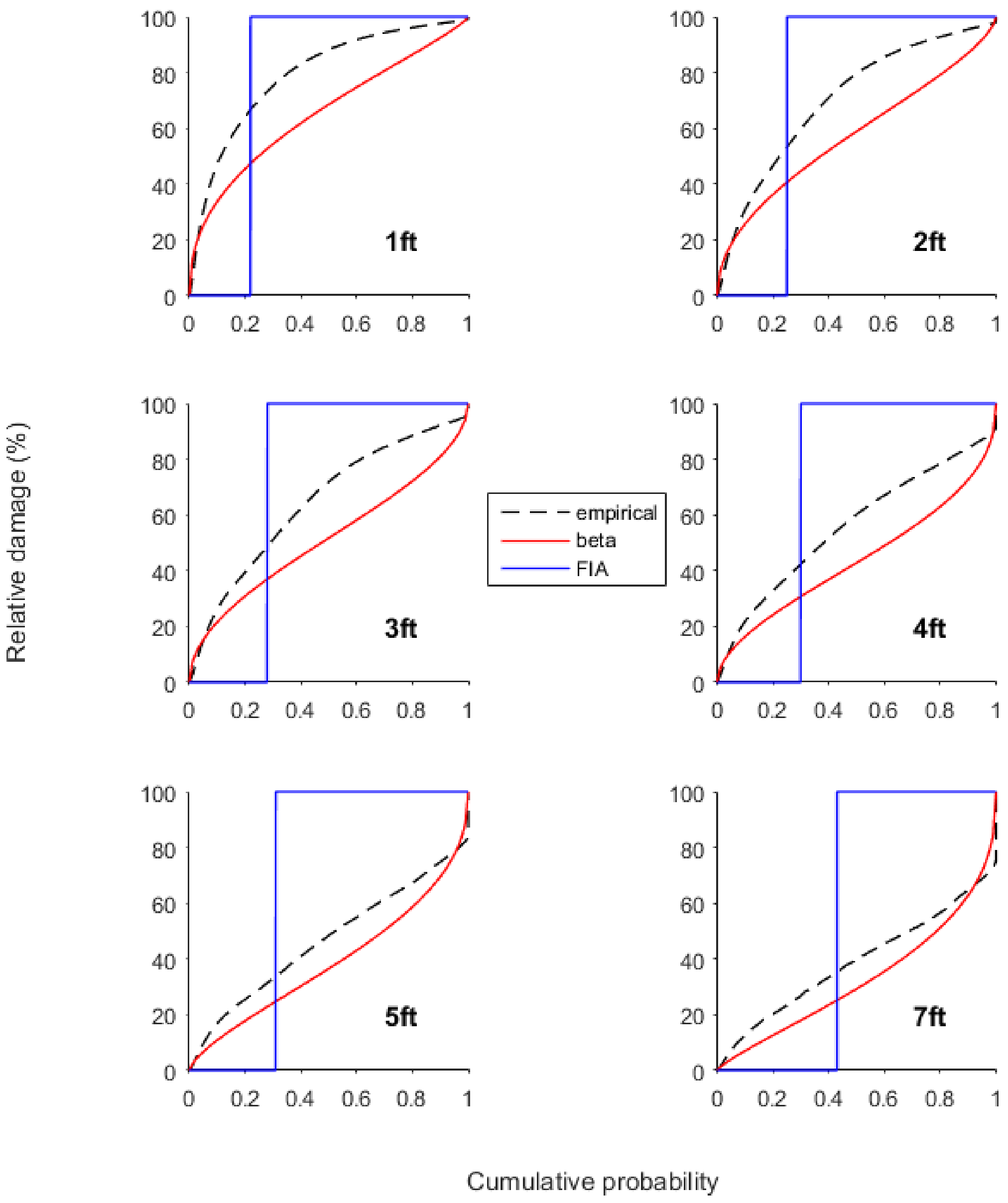

Supplementary Figure 3. Cumulative distribution function of relative damage at six depth increments. The dashed lines represent empirical distributions of damages recorded in the NFIP claims database; the red lines are those of samples from the beta distributions

(Supplementary Figure 1); the blue lines are those of the Federal Insurance Agency (FIA) depth–damage curve (Figure 1).

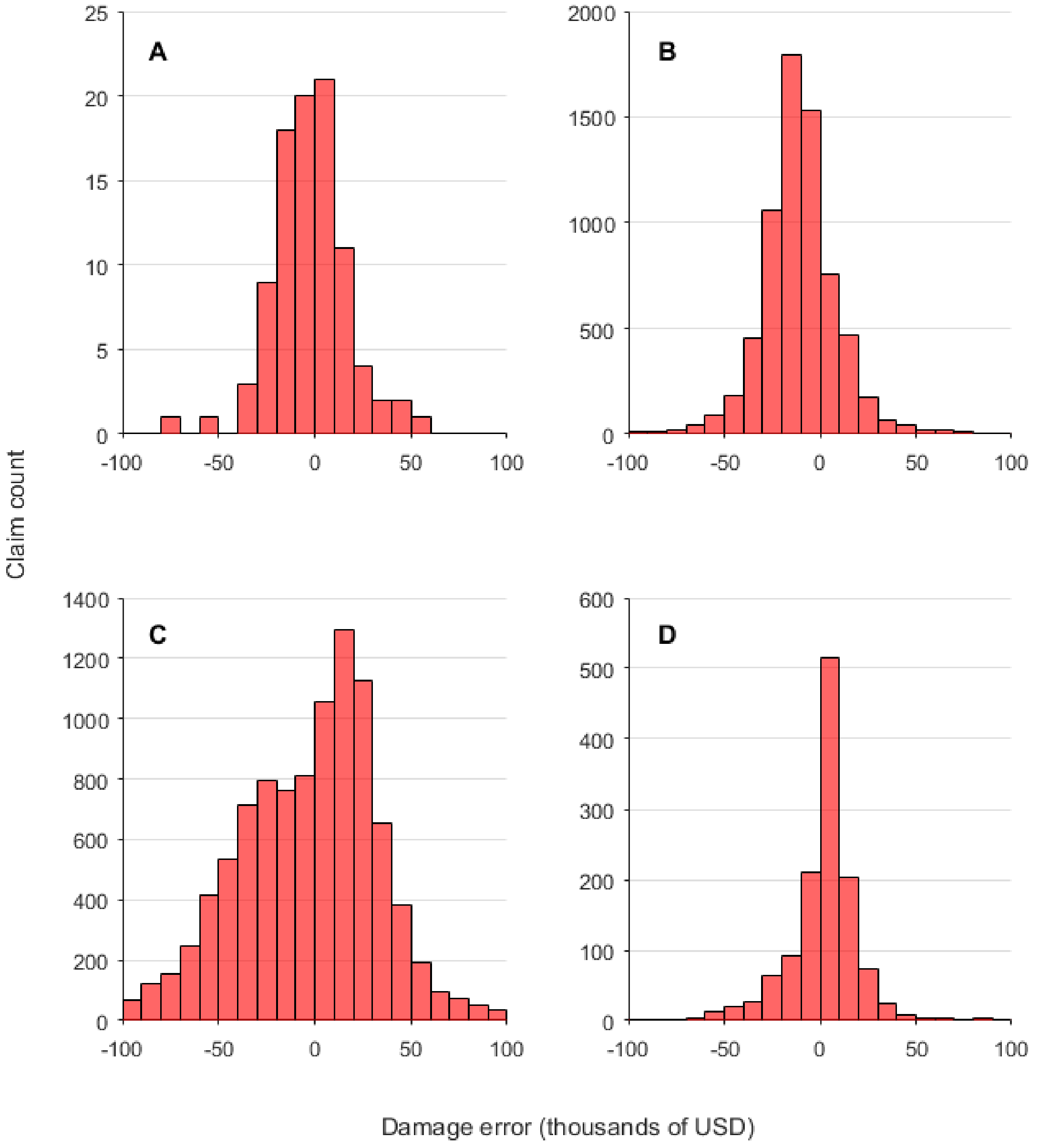

Supplementary Figure 4. USACE curve damage estimation errors for historic flood events. The distribution of errors in building-level loss calculated using a USACE curve compared to the recorded NFIP damage for (**A**) the Chicago 1996 flood event, (**B**) Tropical Storm Allison, (**C**) Hurricane Ike, and (**D**) Hurricane Isabel. Claims are isolated based on whether the zip

code fell within the considered USACE district and if the date of loss was during the considered event.

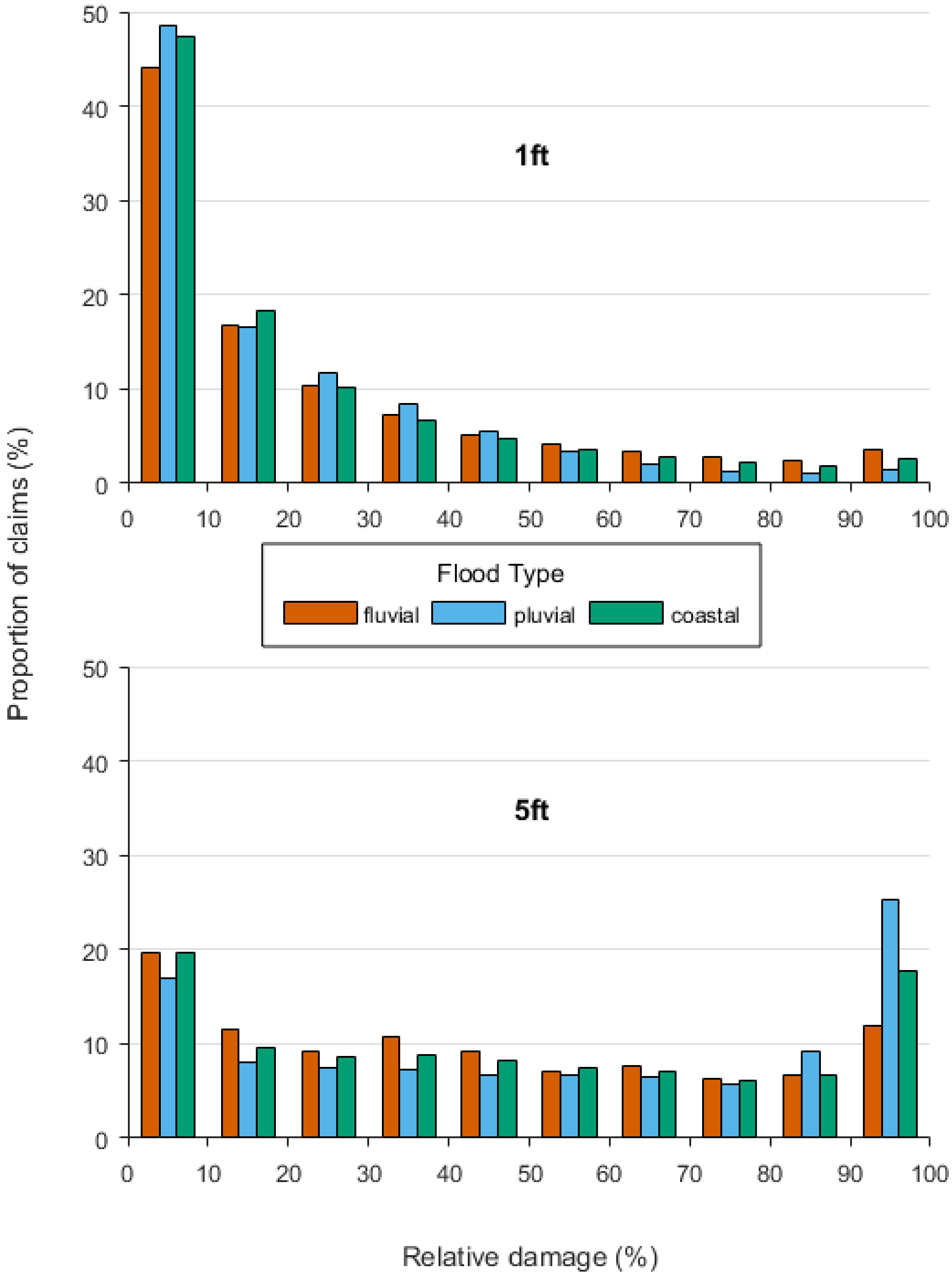

Supplementary Figure 5. Relative damage to one-story residential buildings with no basement stratified by flood type. Sample water depths are illustrated here: 1-foot (upper panel) and 5-feet (lower panel) of inundation.

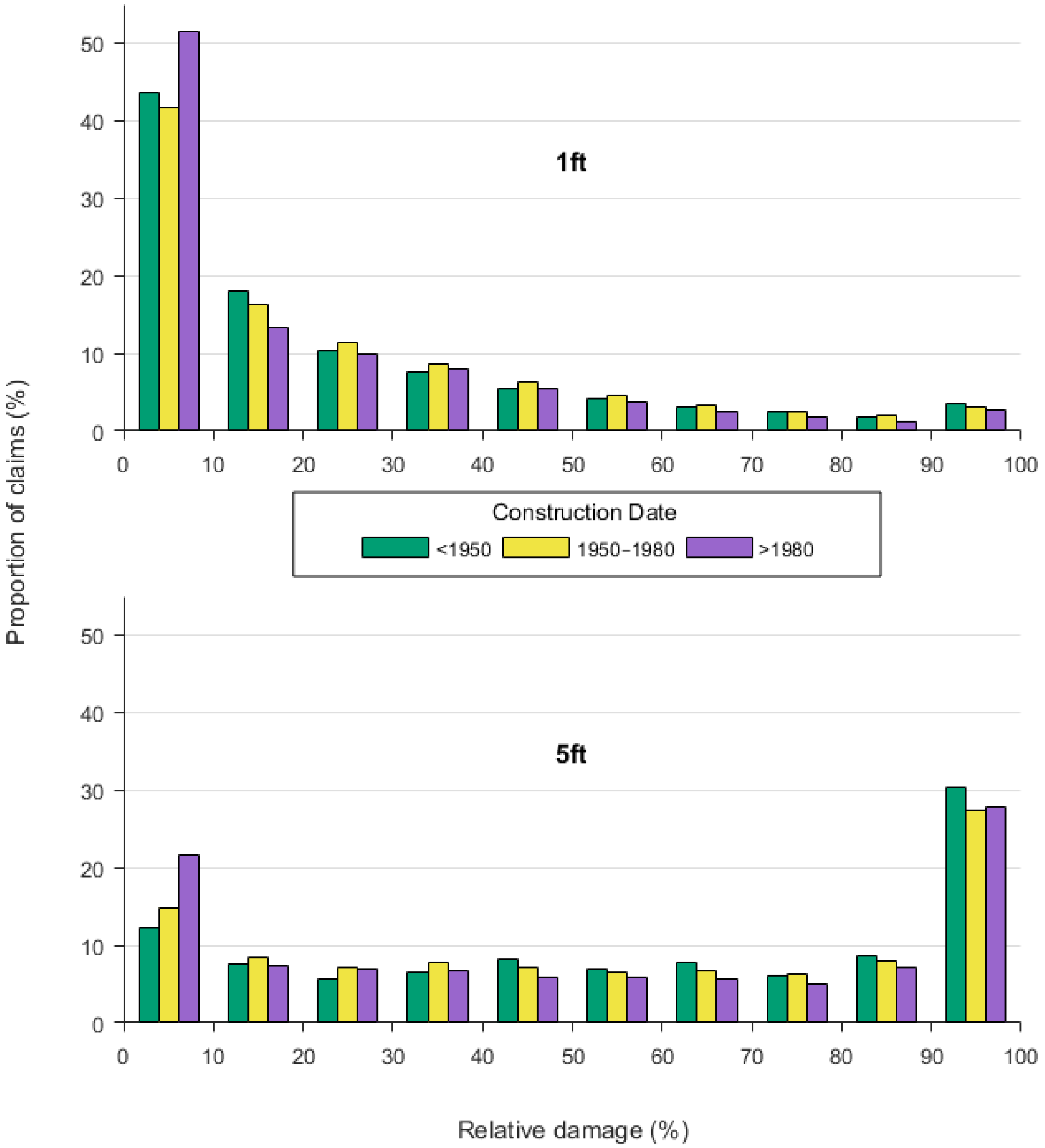

Supplementary Figure 6. Relative damage to different ages of one-story residential buildings with no basement. Sample water depths are illustrated here: 1-foot (upper panel) and 5-feet (lower panel) of inundation.

SUPPLEMENTARY TABLES

	Beta distribution	FIA curve
1-foot	0.78	0.45
2-foot	0.91	0.52
3-foot	0.95	0.55
4-foot	0.99	0.55
5-foot	1.00	0.56
7-foot	1.00	0.69

Supplementary Table 1. R^2 values indicating the degree of similarity of samples from the beta distributions (Supplementary Figure 1) and the Federal Insurance Agency (FIA) depth–damage curve to the empirical distribution of relative damages from the NFIP claims record.

	Number of claims analyzed	Total damage (NFIP) ($)	Total damage (FIA) ($)	Aggregate error (%)	Mean damage (NFIP) ($)	Mean error ($)	Mean absolute error ($)	Coefficient of determination
Total	378,906	10,631,459,512	10,185,079,552	–4.2	28,058	–1178	23,669	–0.04
8-foot	12,570	910,886,383	691,675,194	–24.1	72,465	–17,439	47,529	–0.28
7-foot	9797	709,187,233	530,522,172	–25.2	72,388	–18,236	46,109	–0.12
5-foot	18,458	1,097,904,596	692,411,128	–36.9	59,481	–21,968	42,312	–0.07
4-foot	29,319	1,350,039,478	1,027,811,546	–23.9	46,047	–10,990	34,741	–0.06
3-foot	40,179	1,345,145,452	1,213,780,112	–9.8	33,479	–3270	26,403	–0.08
2-foot	69,593	1,828,027,257	1,782,565,624	–2.5	26,267	–653	21,639	–0.11
1-foot	198,990	3,390,269,113	4,246,313,776	25.3	17,037	4302	17,854	–0.44

Supplementary Table 2. Comparison of damages recorded for all considered claims* and those calculated when using the Federal Insurance Agency (FIA) depth–damage curve nationwide. Negative (positive) errors indicate underprediction (overprediction) by the FIA curve. The coefficient of determination is described in equation 1.

*claims on one-story residential buildings with no basement where depth, structure value and damage were recorded

	Chicago (July 1996)	Tropical Storm Allison (June 2001)	Hurricane Ike (September 2008)	Hurricane Isabel (September 2003)
USACE curve applied	Chicago	Galveston	Galveston	Wilmington
Number of claims analyzed	94	6832	9937	1276
Total damage (NFIP) ($)	2,073,463	230,236,203	449,788,114	17,378,373
Total damage (USACE) ($)	2,333,946	162,159,274	446,963,717	20,267,556
Aggregate error (%)	12.6	–30.6	–0.6	16.6
Mean damage (NFIP) ($)	22,058	33,700	45,264	13,619
Mean error ($)	2771	–9964	–284	2264
Mean absolute error ($)	19,441	19,300	34,213	13,707
Coefficient of determination	–7.95	–0.40	–0.40	–0.44

Supplementary Table 3. Comparison of recorded event damages in the NFIP claims database and those simulated using USACE curves. Each column represents an historic flood event. Negative (positive) errors indicate underprediction (overprediction) by the FIA curve. The coefficient of determination is described in equation 1.

SUPPLEMENTARY NOTE 1: VERIFICATION OF THE BETA DISTRIBUTIONS

The shape of the relative damage distribution at each depth increment (Figure 2) can be described formulaically by the beta distribution (Supplementary Figure 1). Defined in the interval [0,1], the beta distribution therefore ensures relative damages do not fall outside of the 0–100% range and adequately captures the distinctive bimodal shape of depth–damage: where relative damages are concentrated at the minimum and maximum extremities of the spectrum. Supplementary Figure 2A illustrates the correspondence between the empirical and beta distributions, as do the cumulative distribution functions in Supplementary Figure 3. Supplementary Table 1 quantifies this close match with R^2 values approaching 1. Supplementary Figure 2B, the blue lines in Supplementary Figure 3 and the values in Supplementary Table 1 serve to illustrate the fallacy in the use of a one-to-one depth–damage function (in this case, the commonly-used FIA curve) in capturing the empirical spread evident in the NFIP claims.
